# Isolation of nuclear proteins from flax (*Linum usitatissimum *L.) seed coats for gene expression regulation studies

**DOI:** 10.1186/1756-0500-5-15

**Published:** 2012-01-09

**Authors:** Sullivan Renouard, Corbin Cyrielle, Tatiana Lopez, Frédéric Lamblin, Eric Lainé, Christophe Hano

**Affiliations:** 1 Laboratoire de Biologie des Ligneux et des Grandes Cultures UPRES EA 1207, Université d'Orléans, Equipe Lignanes des Linacées, Antenne Scientifique Universitaire de Chartres, 21 rue de Loigny la Bataille F-28000 Chartres, France; 2 Laboratoire de Phytotechnologie EA 3900 BioPI, Faculté de Pharmacie, Université de Picardie Jules Verne, 1 rue des Louvels F80037 Amiens, France

**Keywords:** Flax, Gene expression, Mucilage, Nuclear proteins, Phenolics, Seed coat

## Abstract

**Background:**

While seed biology is well characterized and numerous studies have focused on this subject over the past years, the regulation of seed coat development and metabolism is for the most part still non-elucidated. It is well known that the seed coat has an essential role in seed development and its features are associated with important agronomical traits. It also constitutes a rich source of valuable compounds such as pharmaceuticals. Most of the cell genetic material is contained in the nucleus; therefore nuclear proteins constitute a major actor for gene expression regulation. Isolation of nuclear proteins responsible for specific seed coat expression is an important prerequisite for understanding seed coat metabolism and development. The extraction of nuclear proteins may be problematic due to the presence of specific components that can interfere with the extraction process. The seed coat is a rich source of mucilage and phenolics, which are good examples of these hindering compounds.

**Findings:**

In the present study, we propose an optimized nuclear protein extraction protocol able to provide nuclear proteins from flax seed coat without contaminants and sufficient yield and quality for their use in transcriptional gene expression regulation by gel shift experiments.

**Conclusions:**

Routinely, around 250 μg of nuclear proteins per gram of fresh weight were extracted from immature flax seed coats. The isolation protocol described hereafter may serve as an effective tool for gene expression regulation and seed coat-focused proteomics studies.

## Background

The seed coat plays a crucial role for seed protection against biotic and abiotic stress and has an impact on embryo development, seed dormancy and germination [1]. The seed coat also constitutes a rich source of valuable compounds such as pharmaceuticals, and their features are associated with important agronomical traits [1,2]. Obviously, during the last decade, technologies such as genomics, proteomics and metabolomics applied to the *Arabidopsis thaliana *model have allowed deeper understanding in seed biology [2]. Our understanding of seed coat development and metabolism has taken advantage of this acceleration of knowledge but has not provided enough information about specific biosynthetic pathways to many of our crops [1]. For instance, flax seed coat constitutes a model for the biosynthesis of lignans (diphenolic compounds with high potential for pharmaceutical or cosmetic industries [3,4]) while *A. thaliana *seeds are not known to produce these compounds. Another important example is the accumulation of specific anti-nutritional factors in canola seed coats that require modifications to improve meal quality [5]. In both examples, our knowledge of seed coat biology is still too limited to take advantage of valuable compounds or to improve agronomical quality [1].

Isolation and identification of transcription factors responsible for seed coat specific expression are pre-requisites for the understanding of seed coat development and metabolism regulation. Transcription factors represent only 0.001 to 0.01% of the total cellular protein content and their extraction could be a great challenge [6]. Moreover, compared to other organisms, plants are generally more problematic for protein extraction because they contain high levels of proteases and interfering compounds that can both hinder extraction itself, DNA binding experiments or gel-based separation [7]. Despite the availability of commercial kits and published protocols it is well known that in most cases the extraction procedure must be optimized for each plant species, tissue, or cell compartment [7,8]. The seed coat generally harbors high quantities of interfering compounds such as polyphenols, mucilage, starch and lipid derivatives [1] that can severely affect the performance of protein extraction. Phenolic compounds can build irreversible complexes with proteins and it has been shown that oxidation of phenolics by oxidases and peroxidases can cause streaking and generate artefactual spots on 2D electrophoresis gels [7]. The presence of mucilage may also hinder the separation of proteins due to their swelling in aqueous medium [9]. So far, no protocol published has been designed for the extraction of nuclear proteins from seed coat.

The aim of this study was to extract DNA binding nuclear proteins suitable for gene expression study by gel shift experiments using seed coats of immature flaxseeds as starting material. Gel shift assay is the first step of transcription factor study and this method has been widely used in the study of sequence-specific DNA-binding proteins such as transcription factors [6]. The assay relies on the ability for a protein to bind a labeled DNA fragment in vitro, followed by electrophoretic separation of DNA-proteins complexes that migrate more slowly than free DNA fragments. The key to success of gel shift assay relies on the availability of a sufficient amount of high-quality nuclear protein extracts containing native transcription factors. Flax seed coat is rich in both mucilage and phenolics and therefore constitutes a good example of the presence of these compounds that can interfere with the extraction process. Established methods (commercial and published protocols) have failed to provide suitable nuclear proteins from seeds for gel shift assay. In the present study, we describe an optimized and efficient protocol for the extraction of nuclear proteins from flax seed coat, compare it to other methods and discuss the critical steps. Given the fact that, in a number of plant species, seed coats contain either high mucilage or phenolic contents (e.g. in *Brassicaceae*, *Solanaceae *and *Linaceae *species), the isolation protocol described below may serve as an effective tool for gene expression regulation and proteomics focusing on seed coat in these species.

## Methods

### Plant material

**Figure 1:**
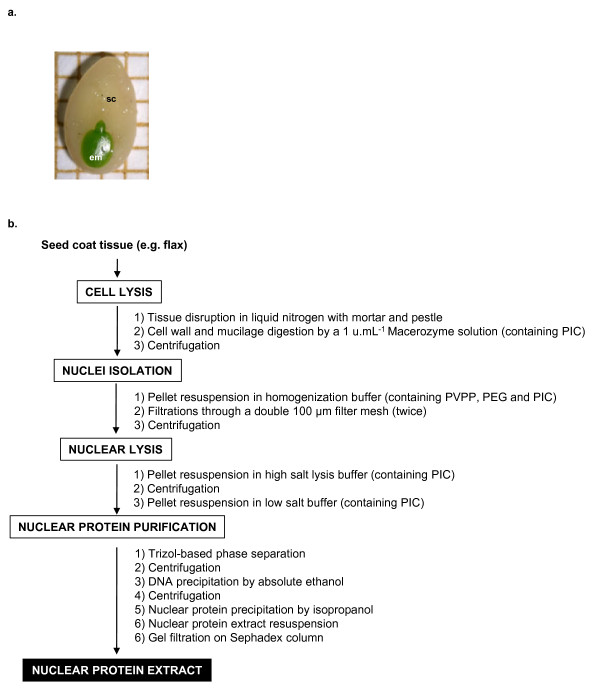
**a Morphology of dissected flax seedcoat (sc) and embryo (em) at developmental stage S2 (Torpedo stage), 16 days after flowering, square representing 1 mm^2^; b**. Workflow of the optimized protocol for extraction of an active nuclear protein fraction from flax seed coat. (For more details see Methods).

*Linum usitatissimum *L. (linseed cultivar Barbara) immature seed coats (development stage S2 [3] (torpedo stage, 16 days after flowering), Figure 1a) were used as starting material.

### Commercial or published methods for nuclear proteins extraction

One commercial kit (CelLytic PN for plant nuclei isolation kit from leaves, Sigma) and two published methods [10,11] were tested as described by manufacturer or following author's recommendations for the extraction of nuclear protein from immature flax seed coats. The commercial kit (Sigma) was designed for nuclear protein extraction from *Arabidopsis thaliana *leaves whereas the two published methods were designed and used respectively for nuclear protein extraction from *Phaseolus vulgaris *whole seeds [10] and maize embryos [11].

### Optimized nuclear protein extraction protocol

The workflow of the optimized protein extraction protocol is summarized in Figure 1b.

#### Cell lysis

Seed coats were ground in liquid nitrogen and incubated in a 100 mM citrate-phosphate pH 4.8 buffer containing 1 unit.mL^-1 ^Macerozyme for 1 h at 25°C.

#### Nuclei isolation

After centrifugation at 8,000 g for 10 min at 4°C, the pellet was resuspended in homogenization buffer pH 8.5 containing 0.44 M sucrose, 2.5% (w/v) Ficoll 400, 5.0% Dextran 40, 25 mM Tris-HCl (pH7.6) 10 mM MgCl_2_, 10 mM β-mercaptoethanol, 0.5% (v/v) Triton X100 and 1% (w/v) PVPP, 2% (w/v) PEG-8000 and 1% (v/v) protease inhibitor cocktail for plants (PIC; Sigma P9599), filtered twice through a double 100 μm filter mesh (Sigma) and centrifuged at 2,000 g for 5 min at 4°C.

#### Nuclear lysis

The pellet was washed twice in a solution containing 25 mM Tris (pH 8.5), 5 mM MgCl_2_, 2% (v/v) glycerol, 10 mM β-mercaptoethanol and 1% (v/v) PIC) and resuspended in high salt buffer (20 mM HEPES (pH 7.5), 25% (v/v) glycerol, 0.42 M NaCl, 1.5 mM MgCl_2_, 0.2 mM EDTA, 0.5 mM DTT and 1% (v/v) PIC), the mixture was left at 4°C for 40 min and occasionally vortexed. Following centrifugation at 8,000 g for 10 min at 4°C, the pellet was resuspended in a low salt buffer (20 mM HEPES (pH7.8), 20 mM KCl, 1.5 mM MgCl_2_, 0.5 mM DTT, 25% glycerol and 1% (v/v) PIC).

#### Nuclear protein purification

Then the resuspended pellet was submitted to a phenol-based purification step: 1 ml of Trizol (guanidium isothiocyanate-phenol-chloroform solution) was added and mixed by pipetting. This solution was then vigorously vortexed for 20 min at 4°C for further nuclear lysis and nuclear proteins solubilization. Following a 5 min incubation at room temperature, 0.2 ml chloroform was added and the mixture was vortexed for 15 s and incubated 5 min to allow phase separation by centrifugation at 12,000 g for 15 min at 4°C. The upper phase containing RNA was removed and the DNA was precipitated by addition of 0.3 ml absolute ethanol. The phenol/ethanol phase was carefully mixed, incubated 3 min at room temperature and centrifuged at 2,000 g for 5 min at 4°C to pellet the remaining DNA. Supernatant was transferred into a new tube for further precipitation of the nuclear proteins by addition of 1.5 ml 100% isopropanol. Following 15 min incubation at room temperature, the nuclear protein were pelleted by centrifugation at 15,000 g for 15 min at 4°C and then resuspended in 1 mL of storage buffer (20 mM Tris pH 7.5, 1 mM EDTA, 1 mM MgCl_2_, 10 mM KCl, 5% glycerol). Residual phenol and salts were removed by gel filtration onto a G-25 Sephadex desalting column.

### Estimation of contamination by cytosolic proteins

The presence of contaminating proteins was estimated using alcohol dehydrogenase (ADH) activity assay [12] as an indication of cytosolic protein presence.

The ADH specific activity was assayed spectrophotometrically with ethanol as substrate. NADH production from NAD was measured by increase in absorbance at 340 nm at 25°C [12]. The reaction mixture contained 0.1 M Tris-HCl (pH 9.0), 2 mmol NAD and 1% (v/v) ethanol in a final volume of 5 ml.

### Protein concentration determination

The protein concentration was determined using a fluorometer and the Quant-iT Protein Assay Kit (Invitrogen) adapted for the Qubit fluorometer according to the manufacturer's protocol.

### Protein western blot and dot blot

Nitrocellulose membranes (BioRad) were prepared and nuclear proteins were applied on the membrane as a dot following manufacturer's recommendation. Nitrocellulose membranes were blocked with 10% nonfat milk in TBS containing 0.1% Tween-20 for 1 h, incubated with a 1:1000 dilution of the commercial antibodies, previously used for plant cellular compartments characterization [8], anti-histone H1 (Sigma, H7665) or anti-actin 2 (Sigma, A2066) antibodies produced in rabbit for 1 h at room temperature and then washed in PBS containing 0.1% Tween-20 three times for 5 min. Blots were incubated with a 1:4000 dilution of the commercial anti-rabbit secondary antibody peroxidase-conjugated (Roche) for 1 h at room temperature, and developed using Lumi-Light Plus POD substrate (Roche). The color intensity was analyzed and quantified by densitometry using a digital camera (AlphaImager 1220; Alpha Innotech Corporation) and densitometry software (Alpha Innotech Corporation).

### Gel shift assay

The band shift assay was performed using the non-radioactive Roche Gel Shift assay kit according to manufacturer's instructions with 10 μg nuclear protein preparation and 10 nM of 5'-Digoxigenin-labeled double stranded oligonucleotide 5'-GAGCATG**CTAACCA**AAATGT-3' containing a MYB binding site (underlined and bold) present in the promoter region of *LuPLR1 *gene [3]. This oligonucleotide was synthesized by Eurofins MWG Operon. The assay was carried out at room temperature for 30 min in a final volume of 25 μL in binding buffer (Roche). The binding reaction products were separated on a nondenaturating 6% acrylamide:bis-acrylamide gel (37.5:1) in a low ionic strength buffer (6.7 mM Tris, pH 7.5; 1 mM EDTA, pH 7.5 and 3.5 mM sodium acetate) at 4°C. The next steps were carried out according to manufacturer's instructions for the Roche Gel Shift assay kit.

### Treatment of data

All data presented in this study were the means of at least three independent replicates ± standard error of the mean. Comparative statistical analyses of groups were performed using Student's *t*-test or one-way analysis of variance according to the data. In figures, the same letter indicates that values are not significantly different. All statistical tests were considered significant at *P *< 0.05.

## Results and discussion

### Evaluation of published methods for nuclear protein extraction

Attempts to use published or commercial protocols on immature flax seed coat for nuclear protein extraction for gene expression regulation were unproductive.

**Figure 2:**
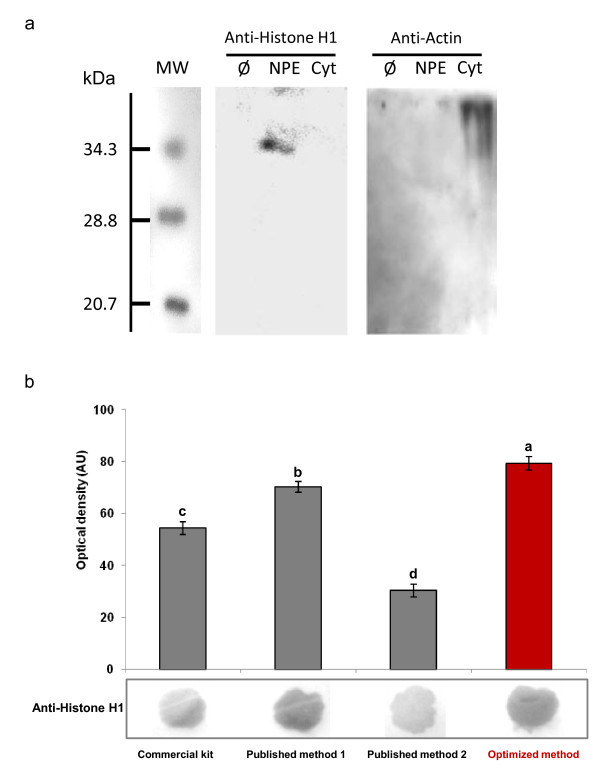
**Protein blots detection of histone H1 and actin 2 a**. Western blot analysis for chemiluminescent detection of histone H1 (around 35 kDa) and actin 2 (around 42 kDa). Nuclear (obtained with the optimized protocol) and cytosolic protein fractions (50 μg) extracted from immature flax seed coats were separated on a 12% SDS-PAGE, transferred to nitrocellulose membrane, and probed with each of the above-noted antibodies. Equal loading of the gels was verified by protein quantization (using fluorescent probe, see Methods) before loading and Coomassie blue staining of the membrane after protein transfer. MW: molecular weight; Ø: Loading buffer without protein (negative control); NPE: nuclear proteins extract (optimized method); Cyt: cytosolic extract (first supernatant, nuclei isolation step). **b**. Protein Dot blot for chemoluminescent detection of histone H1 was performed using nuclear fraction obtained with the different methods. The color intensity was analyzed and quantified by densitometry using a digital camera and densitometry software. The same letter indicates that values are not significantly different (*P *> 0.05). Value is mean ± standard error.

**Figure 3:**
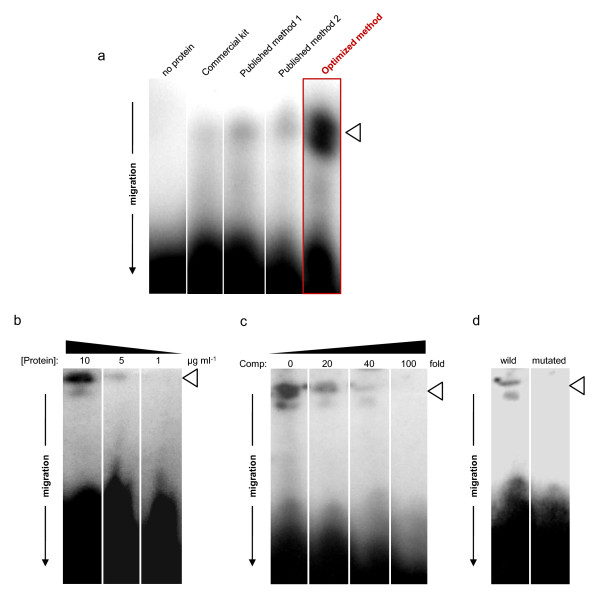
**Gel shift assay for nuclear protein binding on MYB2 site The analysis of the DNA-protein complex by gel shift assay was performed with nuclear extracts using 10 nM DIG-labeled probe fragment with nuclear proteins extracted from immature seed coats from flax (developmental stage 3)**. The gel shift assay was performed in a 6% polyacrylamide gel. White arrowheads show the retarded DNA signals. This Figure is representative of at least 5 independent experiments performed in the same conditions. **a**. Comparative analysis of the DNA-protein complex formation by gel shift assay performed using 10 nM DIG-labeled probe fragment with 10 μg of nuclear protein extracted with the different nuclear protein extraction methods. **b**. Effect of nuclear proteins (obtained with the optimized protocol) concentrations on DNA-binding capacity on the putative MYB2 site. The amount of nuclear proteins added per lane is indicated. **c**. Titration of nuclear proteins (obtained with the optimized protocol) specific affinity for the Dig-labeled MYB2 containing fragment using the same unlabelled fragment at the indicated fold-molar-excess in the presence of 10 μg nuclear proteins extract from immature flax seed coat. **d**. Effect of mutation of the putative MYB2 binding site on DNA-binding capacity of 10 μg nuclear proteins (obtained with the optimized protocol).

The CelLytic PN extraction kit from Sigma, a commercial adaptation of a protocol described to isolate nuclear proteins from *A. thaliana *leaves [12], yielded up to 168.5 μg proteins per gram fresh weight (Table 1). The presence of nuclear proteins in this fraction was confirmed by protein blot using an anti-histone H1 antibody (Figure 2), suggesting that the preparation is enriched in nuclear proteins. But this final extract also appeared contaminated by a non negligible amount of non-nuclear proteins, evidenced by the presence of ADH activity in the final extract (Table 1). The presence of these contaminants could partly explain the fact that this extract failed to produce active DNA binding nuclear protein for gel shift assay. Only a very faint retardation signal was observed (Figure 3).

**Table 1:** Nuclear protein extract concentration and quality estimated by the contamination by non nuclear (cytosolic) fraction nd: not detected; the same letter indicates that values are not significantly different (*P *> 0.05). Value is mean ± standard error

	Commercial kit	**Published method 1 **[10]	**Published method 2 **[11]	Optimized protocol (present study)
**Protein concentration (μg.mg^-1 ^FW)**	168.5 ± 7.2^a^	188.2 ± 6.6^b^	173.8 ± 9.0^b^	**247.8 ± 6.1^c^**

**AdH specific activity (AU.mg^-1 ^FW)**	20.7 ± 3.0^c^	8.8 ± 1.2^bc^	15.9 ± 3.8^c^	**nd^a^**

Similar results were obtained with two published protocols designed specifically for nuclear protein extraction from seed. The first protocol was designed for extraction of nuclear protein from *Phaseolus vulgaris *seed ([10], published method 1) and for the second, from maize embryos ([11], published method 2). Using immature flax seed coat as starting material, these two protocols yielded higher amounts of protein 188.2 and 173.8 μg per gram fresh weight as compared to the result obtained with the commercial kit (Table 1). But as already observed with the commercial protocol, despite the clear presence of nuclear proteins in these extracts (Figure 2), these two extracts were also contaminated by cytoplasmic fraction (Table 1). Gel shift assay with these two extracts produced detectable but very weak retardation signal (Figure 3).

It was frequently observed that in most cases extraction procedure has to be optimized for each plant tissue or cellular compartments [7,13]. The results confirmed this observation. Nuclear protein extraction requires improvements and adaptations for their use with tegument as starting material. The absence of protocols designed for extraction of nuclear proteins from this valuable cell compartment has motivated this study.

### Optimized protocol for nuclear protein extraction from seed coats

The optimized protocol for nuclear proteins extraction from seed coats described in the present study shares common features with established protocols in its general scheme but several critical steps were added or modified to address specific problems encountered during extraction. The general scheme of this optimized protocol for nuclear protein extraction from seed coat is presented in Figure 1b and described in detail in the Methods. It consists of the following steps: 1) cell lysis, 2) nuclei isolation (Additional file 1), 3) nuclear lysis and 4) nuclear protein purification. The critical steps are discussed hereafter.

In the first part of this work, we observed that whatever the protocol used, the final extract had high viscosity and a pale brown coloration (data not shown) that led us to suspect the presence of both mucilage and oxidized phenolics. Seed coats generally contain high quantities of phenolics and mucilage, associated with starch and lipid derivatives [1]. It has been observed that phenolics and mucilage can severely affect the performance of the protein extraction and separation [7,9]. For example, oxidized phenolics can build complexes with proteins and interfere with gel separation but also affect DNA binding capacity of the extracted proteins [7,14]. The use of seed coats as starting material results in a concentration effect of these interfering compounds. Flax seed coats contain huge amounts of phenolic compounds [3] and mucilage [4,9] that are able to hinder the protein extraction. Adaptations described below were performed to minimize problems generated by the presence of these compounds found in high amounts in the seed coats.

Following tissue disruption in liquid nitrogen, a pretreatment with Macerozyme was performed. Macerozyme is an extract of *Rhizopus sp.*, which displays pectinase, cellulase and hemicellulase activities. Indeed, we have observed that Macerozyme (an enzyme mixture commonly used for protoplasts isolation) was able to efficiently degrade the mucilage layer covering flaxseeds (ruthenium red staining disappearance and the reducing sugars release were observed, Additional file 2). This enzymatic mucilage removal allowed the recovery of a less viscous extract and has been previously proved to be beneficial for both proteins [9] and lignans [4] recovery from flaxseed.

To address the problems of viscosity and coloration of the final extract, due the presence of cell wall polysaccharides and phenolics, PEG 8000 and Polyvinylpolypyrrolidone (PVPP) were added in the homogenization buffer. Mucilage is abundant in a number of major crops seed coats belonging to the *Brassicaceae*, *Solanaceae *and *Linaceae *species [1] and in the aerial parts of plants belonging to families such as *Crassulaceae*, *Cactaceae *or *Malvaceae *as well as around the root tips of every plant. PEG 8000 have previously been used to avoid problems linked to the presence of polysaccharides during RNA isolation [3,15]. PVPP is able to trap the phenolic compounds and prevent the formation of complexes with proteins [7,14].

Subsequent to high salt nuclear lysis and protein precipitation, a phenol-based protein purification step using Trizol was essential. It allowed complete removal of the nucleic acids that can interfere during gel shift assay and also of remaining starch granules still present in the extract at this stage. Trizol phase separation has been previously used for nuclear protein extraction from *Xerophyta viscose *[8]. Following this purification step, phenol and salts were then efficiently eliminated by gel filtration performed by Sephadex G25 column. This last purification step was required because phenol has to be totally removed to obtain better DNA-binding capacity of the nuclear protein extract.

It is noteworthy that the protease inhibitor cocktail for plants (PIC) was added in every buffer used. It has the advantage of a wider range of inhibition as compared to the frequently used PMSF (phenylmethanesulfonylfluoride, a serine protease inhibitor). Addition of PIC appears to be essential during Macerozyme digestion, as this cell wall degrading mixture, not being a purified enzyme preparation, can as well display some protease activity.

### Comparison of the optimized protocol with other published methods

The addition of several critical steps for optimal extraction of nuclear protein from seed coat was not detrimental to the protein yield since the 247.8 μg.mg^-1 ^FW of nuclear protein in the final extract was significantly higher than those obtained with the other tested methods (Table 1; a one dimension SDS-PAGE analysis of the nuclear proteins obtained with this optimized method is shown in Additional file 3). This could be due to a better release of protein entrapped into or linked with co-extracted soluble phenolics and/or mucilage, as well as to a less important loss during filtration and centrifugation steps as a consequence of the reduced viscosity of the extract. The presence of nuclear protein was evidenced by protein western and dot blotting performed using an anti-histone H1 antibody (Figure 2a, b), previously used to characterize nuclear protein extracts from the resurrection plant *Xerophyta viscose *[8]. As shown in Figure 2a, histone H1 was presented only in the nuclear protein extract (obtained with the optimized protocol) and not in the cytoplasmic fraction. If all methods resulted in an effective nuclear protein extraction, the yield varied considerably according to the method used and better results were obtained with our optimized protocol (Figure 2b). Beside these quantitative variations, qualitative differences were also observed since ADH activity was undetectable in the final nuclear protein extracts of the optimized protocol, assessing a high purity of the purified nuclear proteins (Table 1). The absence of contamination by non nuclear proteins of the nuclear protein extract obtained with the optimized protocol was further accessed by western blot using an anti-actin 2 antibody: a signal was detected only in the cytosolic fraction and not in the nuclear protein extract (Figure 2a). Finally gel mobility shift experiments were performed using a labeled double stranded DNA fragment containing the binding site and nuclear proteins extracted from maturing flax seed coat (Figure 3). Gel shift experiments clearly evidenced a high and reproducible (n = 6) mobility shift of the DNA fragment containing a MYB2 binding site with nuclear proteins obtained with the optimized protocol (Figure 3a). This interaction was even more pronounced at a higher concentration of nuclear proteins (Figure 3b). Specific competition with the same but unlabelled DNA fragment containing the box fully out-competed the DNA-protein interaction at a 100-fold molar excess (Figure 3c). Moreover, mutation of the putative MYB2 binding site resulted in a complete loss of the retarded signal (Figure 3d). These notable gains were also observed in other experiments using probes containing other fixation sites for seed coat specific transcription factor such as ABRE boxes (abscisic acid responsive element; data not shown). These results highlight the effective binding affinity of the nuclear proteins extracted from flax seed coats using our optimized protocol.

### Conclusion and potential uses

To the best of our knowledge this study describes the first nuclear protein extraction protocol optimized for seed coat. This protocol allows an efficient removal of interfering compounds from the final protein samples, thus providing high quality nuclear protein extracts, suitable for transcriptional gene regulation studies. It was used to obtain nuclear protein from flax seed coat with minimal contamination and sufficient yield and quality for subsequent use in gel shift experiments. Given the fact that a number of plant species harbor either high mucilage or phenolic contents (or both) in their seed coat (e.g. in *Brassicaceae*, *Solanaceae *and *Linaceae *species), this optimized protocol could be of great interest for workers involved in DNA-protein interaction or proteomics studies in these species to obtain information on specific seed coat development and metabolism.

## Competing interests

The authors declare that they have no competing interests.

## Authors' contributions

SR, CC, TL and CH performed the research. FL, EL and CH participated in the experimental design and coordination, and drafted the manuscript. All authors read and approved the final manuscript.

## Supplementary Material

Additional file 1 **Structure of the isolated nuclei from immature flax seed coats a**. Nuclei micrograph under the light microscope. **b**. Detail of one nucleus micrograph under the light microscope. **c**. RNA visualization of Ribogreen-stained nucleus micrograph under fluorescence (excitation 480 nm, emission 520 nm). Nuclei were visualized under the oil immersion lens without and with fluorescence, respectively, using a Leitz-inverted microscope DIAVERT. The bar represent around 15 μm (picture a) and squares represent around 5 μm^2 ^(pictures b and c). Click here for file

Additional file 2 **Ruthenium red staining of the mucilage layer of control and Macerozyme-treated flax seed coats a**. Control seeds were incubated in water. **b**. Seeds were submitted to digestion by Macerozyme solution (1 unit.ml^-1^). After incubation, seeds were stained by 0.5% (w/v) ruthenium red and then rinsed 3 times with distilled water. Arrow indicate mucilage layer (m). Click here for file

Additional file 3 **10% SDS-PAGE of nuclear proteins extracted from immature flax seed coats stained with Coomassie blue**. The results shown are representative of three independent biological replicates. MW: molecular weight; NPE: Nuclear proteins extract obtained using the herein presented optimized method. Click here for file
