# Supplementary figures and images for: Isolation of nuclear proteins from flax (Linum usitatissimum L.) seed coats for gene expression regulation studies
Source: BMC Res Notes. 2012 Jan 9;5:15. doi: 10.1186/1756-0500-5-15 (PMC3285032; doi:10.1186/1756-0500-5-15)

## Slide 1
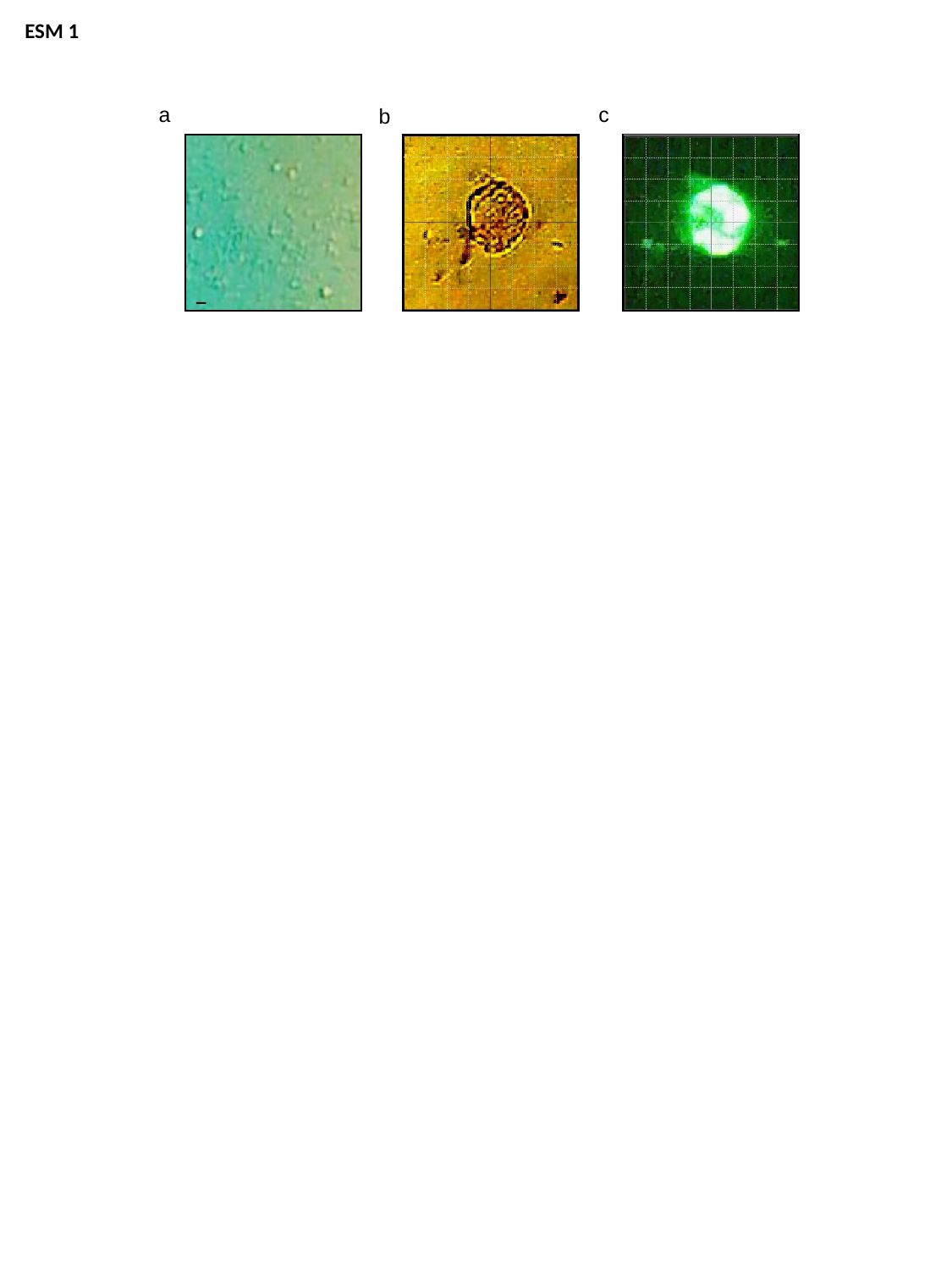

ESM 1
a
c
b

Supplement: Additional file 1 — Structure of the isolated nuclei from immature flax seed coats a. Nuclei micrograph under the light microscope. b. Detail of one nucleus micrograph under the light microscope. c. RNA visualization of Ribogreen-stained nucleus micrograph under fluorescence (excitation 480 nm, emission 520 nm). Nuclei were visualized under the oil immersion lens without and with fluorescence, respectively, using a Leitz-inverted microscope DIAVERT. The bar represent around 15 μm (picture a) and squares represent around 5 μm2 (pictures b and c). [file 1756-0500-5-15-S1.PPT]

## Slide 1
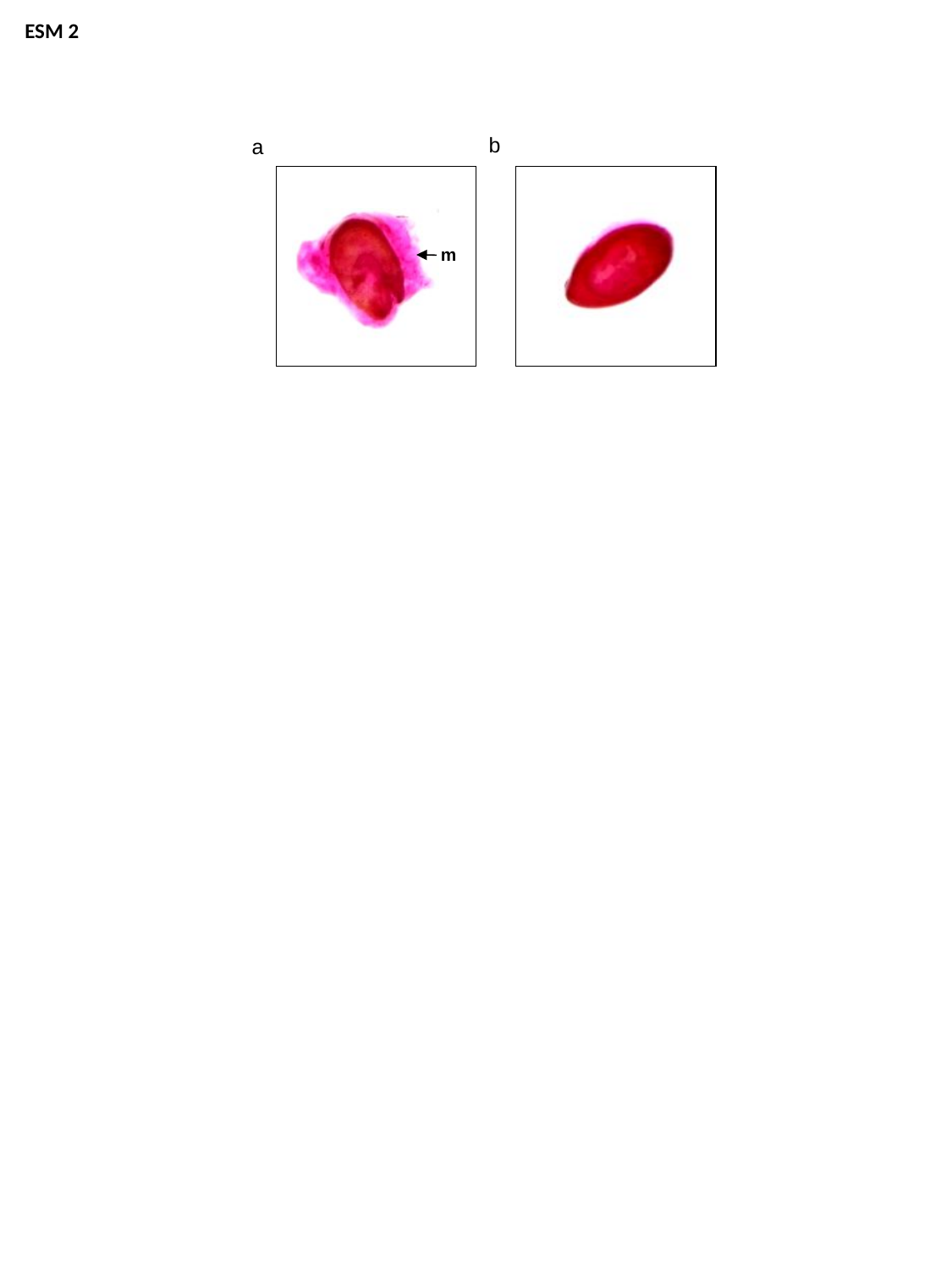

ESM 2
b
a
m

Supplement: Additional file 2 — Ruthenium red staining of the mucilage layer of control and Macerozyme-treated flax seed coats a. Control seeds were incubated in water. b. Seeds were submitted to digestion by Macerozyme solution (1 unit.ml-1). After incubation, seeds were stained by 0.5% (w/v) ruthenium red and then rinsed 3 times with distilled water. Arrow indicate mucilage layer (m). [file 1756-0500-5-15-S2.PPT]

## Slide 1
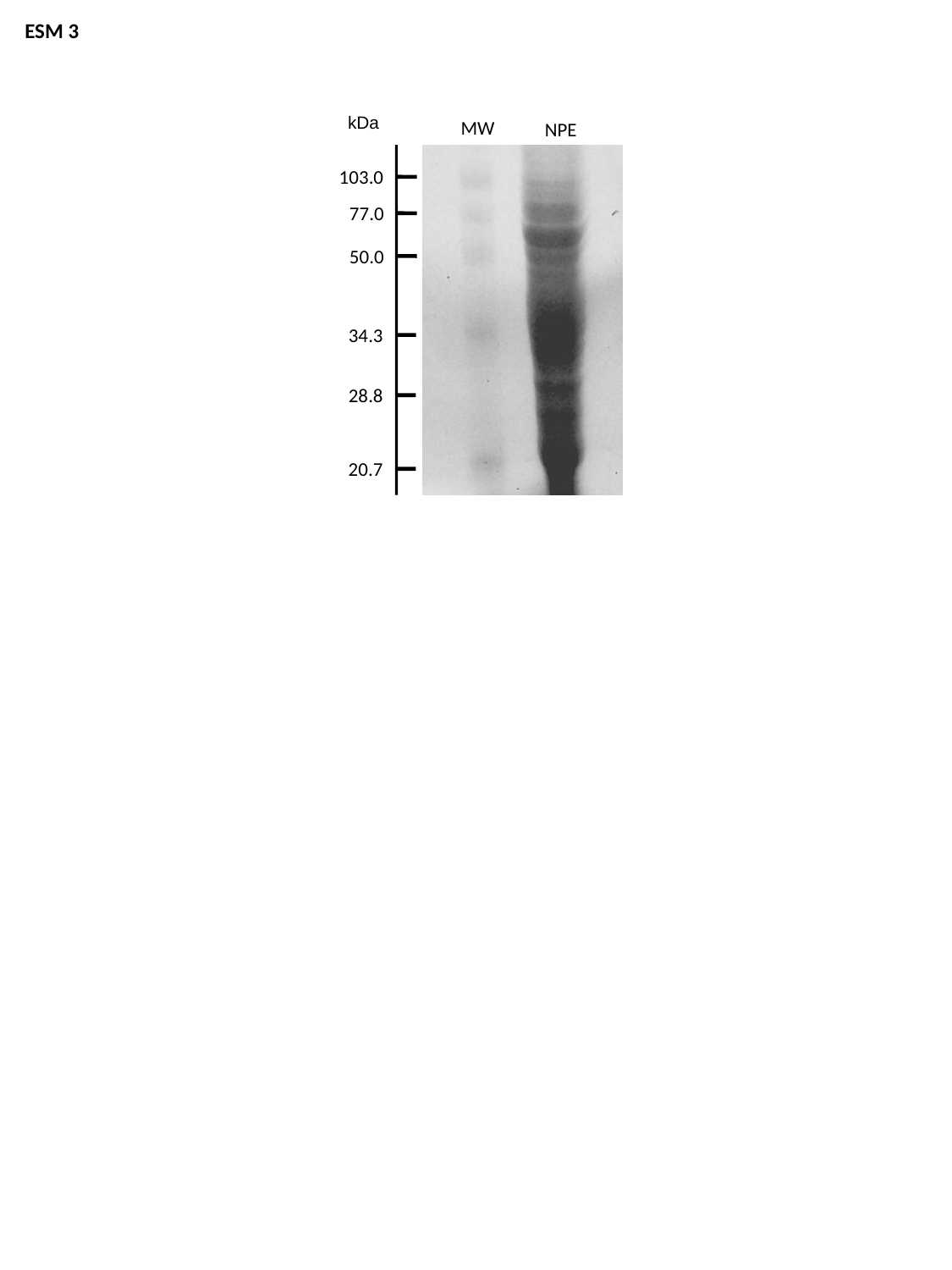

ESM 3
kDa
103.0
77.0
50.0
34.3
28.8
20.7
MW
NPE

Supplement: Additional file 3 — 10% SDS-PAGE of nuclear proteins extracted from immature flax seed coats stained with Coomassie blue. The results shown are representative of three independent biological replicates. MW: molecular weight; NPE: Nuclear proteins extract obtained using the herein presented optimized method. [file 1756-0500-5-15-S3.PPT]
